# Analysis of the watershed social–ecological system trajectory in Copalita-Huatulco, Mexico: The impact of drivers on hydrological ecosystem services

**DOI:** 10.1007/s13280-024-02064-x

**Published:** 2024-09-03

**Authors:** Angel Merlo-Galeazzi, Véronique Sophie Avila-Foucat, María Perevochtchikova

**Affiliations:** 1 Posgrado en Ciencias de la Sostenibilidad, Universidad Nacional Autónoma de México (UNAM), Mexico City, Mexico; 2 Instituto de Investigaciones Económicas, Universidad Nacional Autónoma de México (UNAM), Ciudad Universitaria, Avenida Universidad 3000, Alcaldía Coyoacán, CP 04510 Mexico City, Mexico; 3 Laboratorio Nacional de Resiliencia Costera, UNAM, Mexico City, Mexico; 4 Centro de Estudios Demográficos, Urbanos y Ambientales at El Colegio de México A.C (CEDUA-COLMEX), Carretera Picacho Ajusco 20, Col. Ampliación Fuentes del Pedregal, 14110 Mexico City, Mexico

**Keywords:** Adaptive cycle, Basin, Coupled systems, Historical transformation, Water

## Abstract

In watersheds, which function as social–ecological systems (SESs), hydrological ecosystem services (HESs) are crucial flows connecting natural and social components. The internal and external drivers that shape HESs and watershed SES trajectories create complexities in watershed management. In this study, the adaptive cycle framework was adopted, and a qualitative method is proposed to analyze the impacts of drivers on the HES and the watershed SES trajectory. Empirical evidence from the Copalita-Huatulco watershed in Mexico was used to study the relationships between drivers and HESs. The results reveal changes in regional hydrodynamics due to natural shocks, cumulative social stressors, and SES-related human trajectories. The SES trajectory was in a growth phase when stressors dominated, a collapse when shocks occurred, and a conservation phase when human agency was strong. Understanding the impact of drivers on HESs and their integration into SES trajectories is a key element of HES resilience.

**Supplementary Information:**

The online version contains supplementary material available at 10.1007/s13280-024-02064-x.

## Introduction

Watersheds worldwide face threats from land use changes, climate change, water demand, and pollution, all of which affect their ability to provide ecosystem services and impact the well-being of billions of people (Parsons et al. 2016). Studies assessing these threats have focused primarily on analyzing hydrological processes, spatial modeling, and institutional changes (Tellman et al. 2018; Winkler et al. 2022), but few have approached watersheds as social–ecological systems (SESs) (Wang et al. 2024). From an HES perspective, hydrological ecosystem services (HESs), which are the benefits that people obtain from aquatic environments and the regulation of the water cycle, are crucial since they link social and natural subsystems (Brauman et al. 2007). HESs directly impact human quality of life and well-being by ensuring water quantity and quality for various needs and productive activities (Grizzetti et al. 2016) while sustaining ecological health within watersheds and contributing to long-term sustainability (Ashley et al. 2004).

Studies suggest that analyzing HESs and watersheds from a SES perspective is crucial for successful territorial development and can enhance decision-making capabilities and ensure water sustainability (Gou et al. 2021; Wang et al. 2024). Interest in watershed SES studies, including HES as a flow that interconnects ecological and social components, is growing, as is a focus on ensuring water sustainability at the regional level (Bruckmeier 2016; Wang et al. 2024). Therefore, it is essential to understand how SES drivers of change affect HESs over time and to understand the trajectory of this system to improve management practices that lead to sustainability (Walker et al. 2002; Locatelli et al. 2017). Different frameworks have been used to describe, analyze, and synthesize complex relationships between SES components (Sievers-Glotzbach and Tschersich 2019), demonstrating the human-nature interrelationship and identifying changes (Gunderson et al. 2018).

Nevertheless, research on watershed SES temporal dynamics and interactions has been limited. Cabello et al. (2015) operationalized an SES from a water metabolism framework by applying societal metabolism accounting to land use, human activity, and water use. Mosaffaie et al. (2021) used the driving force–pressure–state–impact–response framework to analyze the environmental issues affecting the Gorganroud watershed. Other examples include qualitative scenario construction (Carpenter et al. 2015) and descriptive analyses of water policies and their impacts on HES access and temporal dynamics (Everard 2020). However, challenges remain in operationalizing SES trajectories at a watershed territorial scale in which the drivers of HES changes over time are identified and quantified (Thonicke et al. 2020).

In particular, this research focuses on coastal SES watersheds since 40% of the global population lives within 100 km of coastlines where human activities tend to intensify and diversify in association with the available natural resources (ONU 2017). At the interface between land and sea, there are multiple socioeconomic and natural drivers, which are often accentuated, especially in Mexico, where the coast is under intense development pressure (Ávila-Foucat et al. 2020). The Copalita-Huatulco watershed (CHW) has national relevance because of the Huatulco Tourist Center (HTC), which is one of the largest tourist centers in Mexico; furthermore, it is a significant coffee-growing region that is threatened by the steadily declining availability of HES (Ramírez-León and Merlo-Galeazzi 2023).

Thus, the objective of this work was to develop a theoretical and methodological framework that can identify and measure the main drivers of HESs and their impacts, as these impacts have defined the trajectory of the coastal SES watershed. In this research, the CHW in Mexico was chosen as a case study.

## Framework background

Watersheds are territorial units in which social and ecological subsystems are interdependent and coevolve to form a SES (Grigg 2016). Analyzing watershed dynamics under the SES framework offers a useful tool for understanding their structure and the interactions between their components; it recognizes that humans are an integral part of nature. In addition, the SES lens facilitates the study of resilience, adaptation, and transformability (Walker et al. 2004) and thus contributes to a deeper understanding of watersheds as complex systems.

Watershed structures and dynamics are largely shaped by the HES since hydrological processes occur within them (Hall et al. 2015). Additionally, water is a key element in human–nature interactions and the most fundamental driver of ecological processes (Chapin et al. 2002; Brauman 2015). Therefore, to ensure the delivery of benefits to society, the drivers that influence SES ecological and social components and the dynamics on which HES flows depend should be well understood (Sun et al. 2017).

**Fig. 1:**
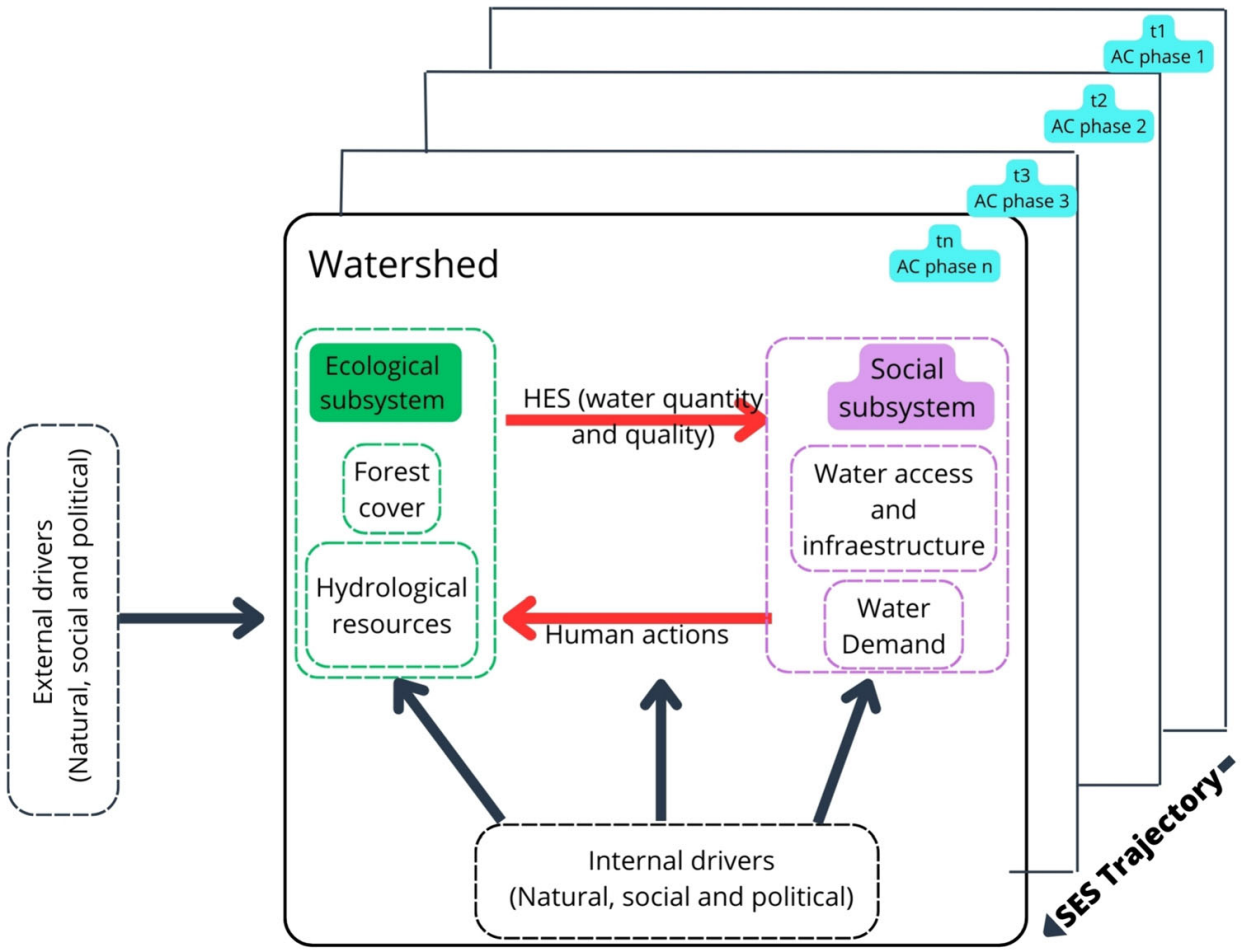
A watershed SES composed of ecological and social subsystems, HESs, and the internal and external drivers that influence them. *Note*: The drivers' characteristics of depth, breadth, and intensity change over time, leading to the emergence of new phases within the adaptive cycle. *Source*: Self-elaboration

HES quality and quantity are influenced by ecological and social subsystem components, which include climate, soils, vegetation, population, or infrastructure (Burkhard et al. 2014; Brauman 2015), and by drivers such as deforestation, urbanization, or climate change, which influence the subsystem components (Hutchins et al. 2018; Bai et al. 2019). The origin of these drivers can be internal or external to the SES. To distinguish the origin of the drivers, the Geist and Lambin (2002) definitions have been adapted as proximal or internal drivers that arise from natural events or human actions that are immediate at the local level with direct impacts on the SES, such as forest fires or changes in land use. On the other hand, the underlying or external drivers are those social, political, or natural forces that support the internal forces and that depend on or are generated outside the SES; these include climatic phenomena, national public policies, or human population dynamics. Both internal and external drivers play crucial roles in ensuring the sustainability of watersheds (Fig. 1).

Drivers that impact watersheds and HESs have been analyzed with spatial models (Martínez-Harms and Balvanera 2012; Nedkov et al. 2015; Li and Quiring 2021; Anjinho et al. 2022; Mokondoko et al. 2022; Wang et al. 2024). Although spatial models based on quantitative data are good decision tools, they do not incorporate variables that are difficult to spatialize, such as the effects of social, political, or economic factors; they also neglect newly emerging drivers that influence the dynamic relationships among SES components (Paudel et al. 2019; Winkler et al. 2022).

Few publications have focused on SES watershed trajectories, including the HES flux. Gunderson et al. (2017) analyzed watershed trajectories on the basis of the role of governance in navigating a changing climate and described the main variables for each period but did not link these changes to drivers over time or to SES components. Wang et al. (2024) proposed an analytical framework by using geographical information systems to visualize interactions between ecological and social systems but considered only direct biophysical changes, such as land use and climate, without political and social drivers. Therefore, to look at driver impacts on SES components and, especially, the HES, the adaptive cycle (AC) could be a useful framework. This approach has been used to study agri-food social–ecological system trajectories. It relates driving forces with adaptive management strategies (Ramírez-León et al. 2023); describes the coevolution of ecosystem services and fisheries (Pérez-Orellana et al. 2020); identifies critical elements of peri-urban wetland resilience to drivers of change (Jiménez et al. 2020); and associates livelihood pathways with environmental changes in coastal lagoons (Thanh et al. 2020).

The AC is a path descriptor consisting of four phases: growth or exploitation (r), conservation (k), collapse (Ω), and reorganization [α]. It reflects the interactions between growth and accumulation and between novelty and renewal, providing insights into persistence and transformation processes in SES dynamics (Holling 2001). The AC is flexible, allowing for the incorporation of internal and external drivers of different natures or origins (Antoni et al. 2019). It also provides insights into dynamic connectivity over time, as it analyzes how systems evolve by incorporating processes of destruction and reorganization (Sundstrom and Allen 2019). By considering these functions, a more comprehensive view of system dynamics emerges; this system links SES organization, resilience, and dynamics, expands the knowledge of variables impacting HESs, and strengthens the socioecological context (Winkler et al. 2022).

For watershed SES trajectories, the AC framework has often been used to describe a narrative path without quantifying the impact of drivers on SES elements (Gunderson et al. 2017; Wang et al. 2024). Tools such as the Leopold matrix can help by summarizing the magnitude of impact over specific SES elements and HESs (Bowd et al. 2015). Drivers are dynamic elements that change in time and space, coevolving and shaping the SES trajectory (Walker et al. 2012). To assess the impacts of drivers, variances in their strength, regional distribution (breath), and temporal patterns (depth) must be considered (Fazey et al. 2018). These concepts are crucial for understanding SES path evolution (Sievers-Glotzbach and Tschersich 2019). Driver properties affect the components of natural and social subsystems and, consequently, the quantity and quality of the HES that connects them. For example, “mature” SES dynamics influenced by “positive” drivers that favor HES provision with specific management interventions can help maintain an SES on a given trajectory, shift it into a different desirable trajectory (Quintas-Soriano et al. 2022), or promote regrowth and reorganization (Chuang et al. 2019).

The contributions of this proposal include the following: (1) an improvement in the incipient understanding of HES as a flow and an important part of the SES trajectory at the watershed scale; (2) the incorporation of a method for quantifying the impacts of various types of drivers on HES and SES trajectories; and (3) an adaptation of the AC framework that utilizes the identification and description of the different phases of SES trajectories to support the above-mentioned method.

## Materials and methods

### Study area: The Copalita-Huatulco SES watershed

**Fig. 2:**
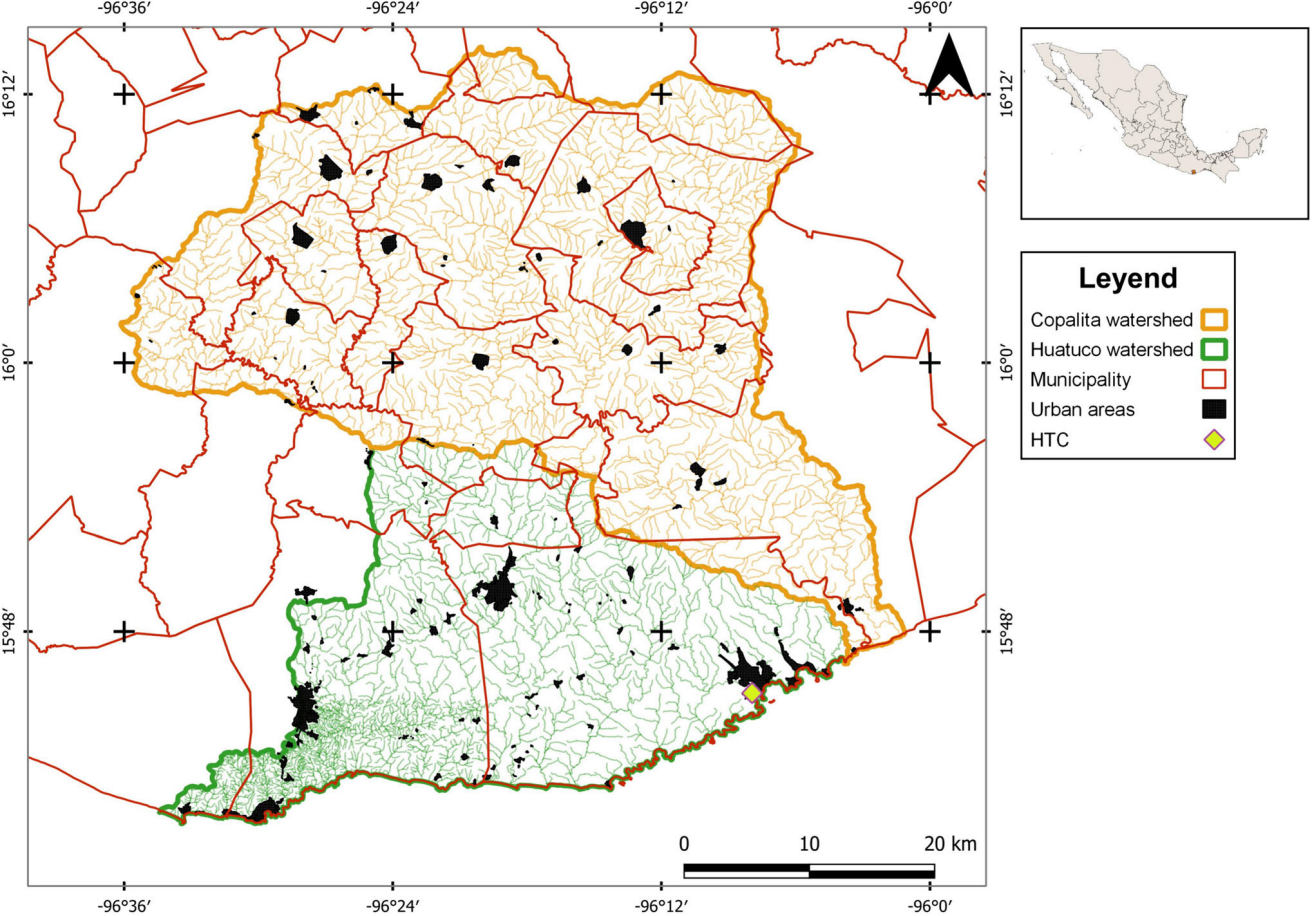
Location of the study area at CHW. *Source*: Self-elaboration

The CHW is a 2366 km^2^ region located between 15° 40′ and 16° 14′ latitude and 96° 00′ and 96° 36′, covering 19 municipalities in Oaxaca state, Mexico (González-Mora et al. 2006) (Fig. 2). Originating from the Sierra Madre del Sur at 3350 masl, this area extends to the Pacific Ocean and has diverse orography, climates, and ecosystems (CONABIO 2013). The upper watershed is dedicated primarily to coffee growing and agriculture, predominantly by indigenous populations, whereas the lower watershed is dominated by tourism activities near the HTC (González et al. 2008; García Alvarado et al. 2017).

#### Ecological subsystem components

Forest cover: The area has a wide range of habitats due to its diverse soils, climate, and intricate topography; the most representative habitats are pine forest (19% of the surface), cloud forest (18%), pine-oak forest (15%), medium subevergreen forest (14%), and medium deciduous forest (11%) (COESFO 2015).

Hydrological resources: The Copalita River is the longest river in the watershed at 78.5 km long, with most of its channels ranging from 16 to 42 km (Escolero 2006). Poor drainage capacity is associated with high slope due to its distance–elevation relationship (Ramírez and Mendoza 2018); moreover, runoff and water infiltration processes are strongly linked with soil, vegetation, and coverage characteristics (Escolero 2006).

#### Social subsystem components

Water access and infrastructure: Despite water availability, the CHW presents an inequitable and insufficient access to water resources. On average, 80% of the population has a piped water supply, although in some municipalities, this percentage decreases to approximately 50% (INEGI 2020). According to the Public Registry of Rights to Water (REPDA, Spanish acronym) of the National Water Commission (CONAGUA, Spanish acronym), the lower areas of the watershed rely primarily on groundwater, whereas the middle and upper areas utilize drains and springs. Therefore, the most developed area, La Crucecita, relies on Copalita river water wells and does not have a district or irrigation unit. Overall, as of 2020, 12 548 749.23 m^3^ of water was being extracted yearly; most of it comes from groundwater extraction (68.5%), followed by surface runoff water (19.17%) and springs (18.37%) (CONAGUA 2020).

Water demand: Regarding water use and demand, a total of 1536 concession titles have been granted to 1479 users (CONAGUA 2020). The National Promotion of Tourism (FONATUR, Spanish acronym) has the largest extraction concession volume with 4 261 372 m^3^, followed by San Pedro Pochutla and Santa María Huatulco municipalities with 1 350 500 and 829 134 m^3^, respectively. With respect to destinations, 36.2% of the volume is for public (urban) use, 35.3% is for agriculture, and 26.9% is for tourism. The greatest water demand occurs in the HTC, which has the largest number of regional inhabitants and tourist activities that require water. Thus, approximately 2.3 million m^3^ per year is consumed in the upper part, and 5.97 million m^3^ is required in the lower part of the HCW (WWF 2004).

#### Hydrological ecosystem services

Water quantity and quality: The annual average precipitation ranges from 1254 to 2722 mm per year, with approximately 90% occurring during the rainy season (Pérez Morga 2013). Runoff water has drinking conditions ranging from slightly acidic (pH 6.3) to slightly alkaline (pH 7.7), with a relative ionic composition dominated by bicarbonates (Mendoza and Andreas 2021). For groundwater, the Huatulco aquifer covers the area and has very good water quality (CONAGUA 2015). Even though water in this region is considered sufficient to meet human needs (CONAGUA 2015), general access to water resources is insufficient and inequitable; furthermore, an average of only 67.8% of households have access to water resources (INEGI 2020).

#### Drivers and watershed SES trajectories

The complexity of the environment and socioeconomy within the SES watershed hinders the identification of the factors that most affect the HES. Hence, the region faces several SES drivers that has slowly and steadily been transformed over the past 40 years. Although the CHW is a subhumid region with relatively low human pressure, the watershed has experienced resource scarcity events, which affect human (economic) activities, mainly tourism (Lozano 2013; Castañeda 2015). Furthermore, since the 1980s, urban development and tourism growth have increased, as have resident and temporary populations, infrastructure, and pressure on resources, which have consequently impacted SES watershed dynamics (Lozano 2013). Moreover, it is unclear which of these drivers impact the HES and how they influence the SES over time.

### Data collection

Semistructured interviews were employed because they are useful for identifying key elements and historical SES dynamics (Andrachuk and Armitage 2015), as well as changes in local communities caused by climate change (Wyllie De Echeverria and Thornton 2019), rapid urban development (Paudel et al. 2019), and public policies (Perevochtchikova and Rojo Negrete 2015). Therefore, as effective tools for documenting both environmental and social forces, they have been used to explain watershed SES trajectory phases (Brattland et al. 2019).

The interviewers were identified via the snowball method (Corbin and Strauss 2006). The first contact was an environmental nongovernmental organization (NGO) representative, who has been working in areas with forest and watershed management for more than 20 years. He helps us identify the first key stakeholders with an in-depth knowledge and understanding of the dynamic and recent history of the watershed. Both local and nonlocal interviewees were considered. Local interviewees were involved in different activities, such as coffee or tourism. Additionally, leaders from different types of local institutions, such as environmental NGOs, and local community leaders were selected. The nonlocal interviewees included representatives of the main Oaxacan and Federal government agencies involved in environmental, water, and forest resource management; representatives of NGOs; and academics. In particular, those individuals who could provide a long-term perspective with at least 20 years in the region that address these issues within their programs and research were selected. Thus, the interviewees represented the different perspectives involved in HES and SES management within the CHW.

Face-to-face interviews (with an average duration of 30 min) were carried out at two time points: the first between June and October 2020 and the second, due to COVID-19 and under the security measures requested by the communities, in November 2020. The objective of the research was explained to the interviewees via a general SES scheme, and authorization was requested to record the conversation under conditions of anonymity and confidentiality. The interviews were divided into four parts: organizational and personal information, drivers’ identification and their relationships with the components of the SES, and periods (Appendix S1). We conducted 27 (12 locals and 15 nonlocals) interviews, which were recorded and transcribed for posterior analysis. The interviewers were NGO members (7), multilevel government representatives (6), coffee growers (6), academics (2), ecotourism cooperatives (2), NGO field technicians (2), silvicultural societies (1), and representative hoteliers (1).

In addition to the interviews, a search for secondary information about the region was carried out using the words “Copalita” and “Huatulco” in the SCOPUS and Google Scholar databases. Additionally, virtual and personalized researchers were visited at the main local universities, and internal reports were requested from the members of the interviewed organizations to enrich the analysis.

### Data analysis

Like Galafassi et al. (2017), a shared narrative was constructed through a hybrid deductive-inductive approach. Themes from interviews and the literature were categorized. This approach enables patterns and incidents that illuminate important dynamics in the emergence of shared meaning and narratives to be identified (Galafassi et al. 2017). Qualitative content analysis was used to classify the collected information into codes as more interpretable units (Kuckartz and Rädiker 2019).

As a first step, drivers were classified into shocks, stressors, or feedback. Shocks were considered sudden, high-magnitude events that can rapidly lead to changes; meanwhile, stressors were slow but steady forces that push a system toward its threshold (Walker et al. 2012; Biggs et al. 2018). Feedback was defined as all those drivers mentioned as positive for one or more SES component statuses; examples include government programs that improve access to infrastructure or human actions, such as reforestation, increase forest cover.

Additionally, drivers were classified as natural, social, or political and coded as internal or external on the basis of an adapted version of the Geist and Lambin (2002) proposal. In these terms, drivers related to the extension of infrastructure (road, urban, public services, etc.); human activities (agriculture, tourism, extraction); community-based strategies (conservation, forest/water management); and natural phenomena whose development is within the physical limits of the SES (fires, plagues) were considered internal. On the other hand, drivers related to demographic factors (population density, migration, etc.); policy and institutional factors of state, national, or international origin (government programs, NGO programs, formal policies, etc.); and natural phenomena of origin or development outside the physical limits of the SES (climate change, hurricanes, etc.) were considered external.

Second, the drivers were related to the HES (water quantity and quality), which determines their relevance. The time periods of drivers were identified through the impacts of key events or “tipping points” that caused discontinuities in the system's history, as determined by stakeholder knowledge and perceptions (Brattland et al. 2019; Bruley et al. 2021). Watershed SES components were established for each period. Finally, each driver-component relation was coded with value impacts (Table 1) according to the perceived magnitude of driver characteristics (Fazey et al. 2018), as follows: (1) breadth (physical space of influence, e.g., the entire watershed or a portion of it), (2) depth temporality of the effects caused (for example, short-term or permanent), and (3) intensity (magnitude). For the driver impact score, an adapted Leopold matrix (Leopold et al. 1971) was used to determine three ranges of impact: 1 for the lowest, 5 for the medium, and 10 for the highest (Table 1); negative scores were applied for stressors and shocks and positive values for feedbacks.

**Table 1:** Score criteria according to the magnitude of the drivers’ impact characteristics. *Source*: Self-elaboration

Driver impact characteristic	Score criteria according to the magnitude of the drivers’ characteristic		
	1 point	5 points	10 points
Breadth	Punctual. The impact is geographically localized in a small area	Local. The impact occurs in several areas but no more than 50%	Watershed. The driver affects all or almost all the watershed
Depth	Temporary. The impacts disappear completely if the driver disappears	Accumulative. The impacts would be partially reversible even though the driver is no longer	Permanent. The impacts are irreversible
Intensity	Low intensity perceived	Medium intensity perceived	High intensity perceived

### Adaptive cycle phase description

Each period identified was characterized in terms of the type of drivers and their impacts on the HES through the total value scores. For this purpose, the net driver impact value (DI_ct_) of each driver for each period was calculated via an adaptation of Leopold's impact (Leopold et al. 1971) formula (Eq. 1).

1 $${\text{DI}}_{{{\mathrm{ct}}}} \, = \,{\text{Br}}_{{{\mathrm{ct}}}} \, + \,{\text{Dp}}_{{{\mathrm{ct}}}} \, + \,I_{{{\text{ct}}}}$$

where DI_ct_ = driver’s impact over one component at period 1; Br_ct_ = impact breadth over one component at period 1; Dp_ct_ = impact depth over one component at period 1; *I*_ct_ = driver intensity over one component at period 1.

The DI_ct_ value, which represents the impact of drivers on each component, is determined by three impact characteristics. Each feature is awarded a maximum impact score of ± 10 points. As a result, the DI_ct_ value can range from − 30 (indicating high negative impact drivers) to 30 (indicating drivers with high positive impact).

Equation (1) was also used to assess the effects of all the drivers identified in all the SES components. For example, Hurricane Paulina’s depth had a permanent effect on forest cover and hydrological resources but had a medium impact on access infrastructure. Additionally, drivers may have positive impacts on one component but negative impacts on another. For example, coffee growing has a permanent positive impact on forest cover but a low-depth negative impact on water quality.

Finally, the total driver impact (TDI) score (Eq. 2) was obtained as the sum of the DI_ct_ values for each of the SES components, estimated via Eq. 1:

2 $${\text{TDI}}\, = \,\sum {\text{DI}}_{{{\mathrm{C}}1}} \, + \,{\text{DI}}_{{{\mathrm{C}}2}} \, + \, \cdots {\text{DI}}_{{{\mathrm{C}}nI_{{{\mathrm{ct}}}} }}$$

where DI_c1_ = driver’s impact over the first component at period 1; DI_c2_ = driver’s impact over the second component at period 1; DI_c*n*_ = driver’s impact over the* n* component at period 1.

Once each identified period was characterized in terms of the type of drivers and their impacts through the TDI, the phases of the AC were subsequently identified for SES trajectory analysis (Table 2). For example, the transition from phase k to Ω was identified via interviews’ perceptions of DI on the SES components and confirmed by scores and evidence of negative or positive senses. Finally, Sankey diagrams were created, and the DI_ct_ values were used to illustrate the relationships between the drivers of the HES of each phase and the SES components. In this manner, AC phases and their corresponding driver-HES-SES relationships were evaluated via qualitative data.

**Table 2:** Characteristics of the drivers for determining the AC phases. *Source*: Self-elaboration

AC phases	AC phase description
Growth-r	Initial phase of the AC and initial score
Conservation-k	There are a greater number of positive drivers, so the SES is in a relative balance
Collapse-Ω	There is the presence of shocks or some broad-spectrum stressor that generates negatives impacts
Reorganization-α	The new conditions are settled, new stressors appear, there is no shock present, and feedback is absent or limited. A new *r* score it assigned

Thus, the study identified the general drivers throughout the SES history and classified them according to their nature. These drivers were related to the HES, and their influence on quality and quantity was described. Finally, the SES trajectory path was developed on the basis of drivers and their impact on the HES and other elements of the SES; it is represented on Sankey’s diagrams.

## Results

### Drivers

A total of 27 drivers were identified as impacting the HES and SES components. Among these, the majority (61.9%) of the categorized drivers were internal social drivers (Table 3). To enhance the description of the drivers' influence on the SES components, a comprehensive literature search was conducted (Appendix S2).

**Table 3:** Classification of the drivers described by the interviewees

Drivers types	External drivers	Internal drivers
Natural	Climate change, Earthquakes, Hurricane Paulina	Bore worm, Fire
Social	Population growth, Tourism growth	Alternative tourism, Aquaculture, Coffee growth, gravel extraction, Huatulco Tourism complex (settlement), Hydraulic infrastructure, Intensive agriculture, Livestock, Roads, Sewage infrastructure, Slash and burn agriculture, social organization, urbanization,
Political	Nongovernmental Organizations programs, CONANP programs, Other federal programs, Payment for ecosystem services, Ramsar declaration, Watershed commit, Huatulco National Park	Community Systems for protected natural areas (CSAP)

### Drivers’ impact on HESs

In general, the HES of water quality is considered to have a greater number of factors of change than does the HES of water quantity. However, among the factors associated with relevant changes in the SES, those related to HES quantity were associated with hurricanes, earthquakes, and climate change.

Hurricane Paulina was described as a milestone in the region because of forest cover loss, which affects water resources, and HES flux changes (river and stream courses), which significantly decreased water quantity and distribution, as mentioned below:

*… from Paulina, it began to make those changes (hydrological), and, strangely, they were all buried; that is, the water was powerful, and many streams and springs were buried… I have memories of Paulina reaching some towns beyond mine, and people said: Paulina also took my river. Where is the pool where I washed? Where is the pool where my children bathed? Everything is sand.* Coffee farmer 1.

Similarly, earthquakes have had direct and indirect impacts associated with forest cover loss because of landslides and hydrological flux changes caused by the decrease or disappearance of water springs, creating challenges for inhabitants who must adapt their infrastructure to new conditions, as noted in the following comments:

*The earthquakes happen here very often; they pass, and I imagine that the water also sinks every time the earthquakes happen.* Coffee farmer 2.

Climate change was associated with an increase in regional temperatures and rainy patterns, which have been perceived since 2000, and was considered to have an impact on water quantity, which has been documented in regional reports (Lozano 2013) and noted by locals:

*It is interesting because it no longer rains as before. It seems that at the end of the year, the same number of millimeters, but how does the rain behave? You have torrential rains for a couple of days that destroy everything, and then very long periods with nothing; this is not regular rain.* Coffee farmer 2.

Tourism and urbanization create constant direct and indirect pressures within the HES, as they are associated with increasing demands for infrastructure, which has other effects, such as changes in regional hydrodynamics due to the extraction of stone material from rivers, as mentioned below.

*The government, they expand airports and roads, making it (gravel exploitation) necessary perhaps, but… it affects us a lot in terms of rafting activities in the dry season; the rafts no longer pass because they get stuck on the ground when the river dries up.* Ecotourism cooperative.

The positive points associated with HES maintenance include the increase in sewage infrastructure, the implementation of government programs such as payment for environmental services (PES) and the maintenance of the shade coffee system. Coffee produced in the shade favors the maintenance of the environmental conditions, vegetation, and soil that support the forest cover offered by the HES.

*…the coffee-growing method relies on the shade. Therefore, shade-grown coffee growers are allies of conservation because, for the coffee to have that quality, it needs to be shaded from larger angles *Oaxaca Government.

### Adaptive cycle phases

Four periods on the SES trajectory through AC phase analysis were established: (1) the arrival of the tourist center and the establishment of a new regional economic dynamic from 1980 to 1997; (2) a transitional period from 1997 to 2000; (3) a period characterized by the PES program and the emergence of NGO works from 2000 to 2015; and (4) the decline in conservation efforts and the identification of climate change-related factors from 2015 to 2020. The identified AC phases are described below.

#### Growth (r) phase: 1980–1996

**Fig. 3:**
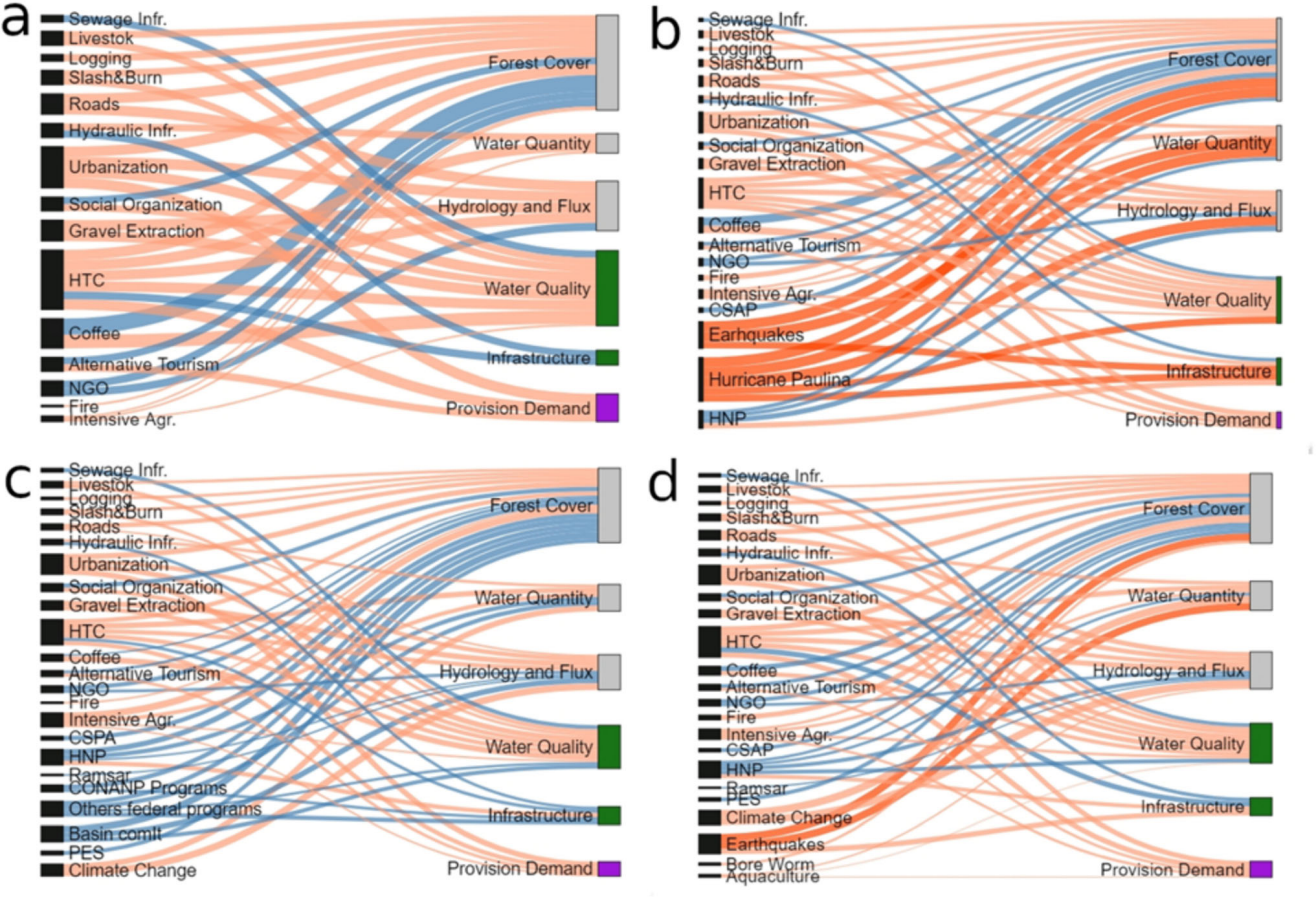
Driver impact on the HES and each SES component for AC phases: *r* phase 1980–1996 (**a**), Ω 1997–2000 (**b**), k 2001–2014 (**c**), and Ω 2015–2020 (**d**). Note: Drivers (in black) impact ecological SES subsystems and component (in gray) and social subcomponents: infrastructure access (in green) and demand (in purple). The thickness of the line is related to the value of the TDI of identified shocks (in orange), stressors (in light orange), and feedback (in blue). A table with each value is presented in Appendix S3. Acronyms: payment for ecosystem services (PES), Huatulco Tourist Center (HTC), Huatulco National Park (HNP), community systems for protected natural areas (CSAP), nongovernmental organization programs (NGO)

This first SES trajectory phase, with a TDI of − 252, was characterized by a combination of stressors that shaped the relationship between human activities and the HES. With the development of tourism in coastal regions, new relationships have formed, and SESs have reorganized (Fig. 3a). In this phase, the established of the HTC was the largest SES event; furthermore, it led to a reorganization of the relationships between the population and HES in the lower watershed, as infrastructure access and the demand for benefits changed. The HTC operation meant an exponential rise in water demand due to the massive flow of tourists, which, according to official data, was 13 850 per year (SECTUR 2019). The new connectivity conditions and development opportunities associated with the HTC increased immigration rates; by 1990, the region’s population was 67 269 inhabitants (INEGI 1990), leading to forest cover loss due to urbanization processes.

Owing to the demand for construction materials, companies have started exploiting river stones, which influence river regional functioning. Population growth, an external driver of increased food demand, has intensified agriculture, logging, and livestock drivers associated with forest cover loss. The slash-and-burn agriculture system is widespread, accounting for approximately 5% of forest loss (Jiménez 2005). On the other hand, coffee production was a relevant positive driver in the region.

For infrastructure access, eight extraction wheels were built on the banks of the Copalita River (Orozco Cervantes 1992), with 56% of the population having hydraulic infrastructure and 23% having sewage infrastructure (INEGI 1990).

#### Collapse (Ω) phase: 1997–2000

This SES trajectory phase had a TDI of − 517, mainly due to the impact of natural external shocks leading to significant ecological changes; moreover, new external institutional drivers occurred, leading to a mixture of collapse and reorganization (Fig. 3b). In this phase, Hurricane Paulina and a strong earthquake struck the region in 1998, with impacts on HES components but also on forest cover and SES hydrological processes.

With respect to internal drivers, some farmers abandoned their coffee crops, and the number of coffee farms began to decrease along with their positive impact on forest cover maintenance. In addition, in 1998, conservation efforts that focused mainly on food security and soil restoration through agroecology and alternative tourism were developed by community organizations, which created community systems for protected natural areas (CSPAs) (González et al. 2008). External drivers such as Huatulco National Park (HNP), which was established in 1998 as a protected natural area, immediately became the most important conservation area for forest cover and hydrological flux processes at the local level (Cid Rodríguez 2006).

#### Conservation (k) phase: 2001–2014

Despite a significant increase in stressors, conservation remained balanced due to the growth of external positive drivers, mainly from government and NGO actions (Fig. 3c). This is reflected in the total impact value, which is − 122. In 2003, 3077 hectares were declared Ramsar (Jiménez 2005), promoting the conservation of regional hydrology in coastal zones. Feedback has also occurred through different instruments implemented by the government. In this sense, the PES program was the public policy instrument with the most significant impact. This program began in 2003 and reached its peak in 2012, with 22 500 hectares (CONAFOR 2019) given for forest conservation, reforestation, monitoring, and firefighting actions in return for economic compensation. Finally, in 2004, the Copalita and Tonameca Watershed Committee formalized its attributes to enable discussions of problems and horizontal decision-making processes among various actors (CONAGUA 2007) and to positively impact several SES components, such as HES quantity, hydrological resources, and infrastructure access. From internal feedbacks, the CSPA was extended significantly by community systems for biodiversity conservation (SICOBI in Spanish acronym) through community management agreements, ecotourism, agroecology, and specialty coffee production (González et al. 2008), with a positive impact on forest cover conservation.

Despite these efforts, the primary stressors in the region intensified, as intensive agriculture and livestock water demand increased by 3% (CONAGUA 2020). In 2010, nearly 16% of the territory was used for slash-and-burn agriculture (Lozano 2013), with a growing impact on forest cover. The HTC has experienced significant growth, with 26 608 tourists arriving (SECTUR 2019), whereas urbanization has increased due to 15% population growth (INEGI 2010). To meet the growing water demands, two new wells were drilled; this drilling affected nearby settlers, whose well water levels decreased.

#### New collapse (Ω) phase: 2015–2020

Recent years have seen a trend toward a new collapse phase of CHW due to the intensification of stressors, shock, and deterioration of previously established positive driver impacts on the HES (Fig. 3d), leading to a TDI score of − 476. A screwworm infestation impacted forest cover in 2015, leading to an increase in logging, forest fires, and slash-and-burn agriculture, as mentioned below:

*In the past two years, a plague has become endemic to the region, posing yet another difficulty. This plague begins to dry out all the trees and contaminate the entire region, and thus far, the only solution is to immediately cut down all the infected trees and burn their branches.* Local NGO.

Intensive agriculture resulted in a significant increase in water demand of 36% in 2019 (CONAGUA 2020), primarily in coastal zones. The total population for 2020 was 132 443 individuals (INEGI 2020), with a growth of 11% relative to that in 2010 and almost double the total population in 1990, indicating increased urbanization and hence water demand, in addition to the 41 988 tourist visits to the HTC per year (SECTUR 2019). The increase in HTC urbanization has meant a greater demand for river gravel extraction, affecting the hydrological functioning and HES flux. According to local testimonies, 20 years of stony material exploitation were reflected in the river's flow width.

In 2019, a strong earthquake had the same effects as those mentioned previously. Furthermore, the effects of climate change have intensified, and water scarcity periods in the tourist area have been recorded more frequently; hotel supplies have been prioritized, leading to water restrictions for villagers, who may spend several days without drinking water (Castañeda 2015). Moreover, storm events linked to the intensification of climate change have led to the loss of infrastructure, which has had to be replaced.

In addition to the increase in the number of stressors, there has been a sharp decrease in positive driver impacts. Beginning in 2015, the financial resources for the watershed committee were reduced, and in 2018, they were eliminated. Finally, the PES program decreased dramatically, comprising less than 2500 hectares in 2019, which was only approximately 10% of its maximum extent in 2012 (CONAFOR 2019).

## Discussion

### Drivers’ impact on the HES

The HES of the study area is influenced by various drivers, including climate and land use changes, similar to those widely reported in other publications (Vignola et al. 2009; Brauman 2015). Therefore, agricultural and urbanization processes directly impact soil properties (Foley et al. 2005; Martínez et al. 2009; Khoie et al. 2023), whereas climate change affects the HES because of temperature increases and rainfall pattern modifications (Hutchins et al. 2018; Bai et al. 2019). The rise in “sun and beach” tourism has increased water demand and led to high water consumption, resulting in inequalities between tourists and residents due to the great differences in their consumption levels (Becken 2014). The proliferation of tourism in coastal watersheds has led to a significant increase in HES impacts, affecting water access and use and demonstrating the role of the HES as a flow that links various components of the SES and as a determinant of its trajectory.

Social and political drivers can have a relevant influence on the regional HES of water quantity and quality and therefore on SES. For example, equal distribution and access to water depend on the capacity to install and operate water infrastructure, which strongly influences water access (Gunderson et al. 2018) and regional development (Molle and Wester 2009). On the other hand, the implementation of public policy instruments can influence ecological conservation (Asbjornsen et al. 2017) and water access rules (Pannu 2012). For example, actors associated with PES programs have positive perceptions of the effects of the program on HESs, in accordance with other authors who reported similar effects of this conservation policy (Rodríguez-Robayo et al. 2016). In parallel, NGO programs play a relevant role in ecological conservation strategies (Keeley et al. 2019) or as entrepreneurs in market-based strategies (Hrabanski et al. 2013), with impacts on SES elements and HES quantity and quality.

Moreover, one of the most relevant findings in this research was the relationships between drivers and HESs, where shocks have long-term effects. Hurricanes and earthquakes were linked to HESs and various components of watershed SESs, such as changes in forest cover and hydrological resources, which pose challenges to communities. Both hurricanes (Her et al. 2018) and earthquakes (Wang and Manga 2009) have been linked to long-term changes in regional dynamics via structural forest damage, pH soil changes, river and stream dynamics, and groundwater levels.

### SES trajectory through AC phases

The SES trajectory of the CHW passed through different AC phases, which can be identified as follows: (1) transition from r to Ω, (2) change from Ω to k, and (3) a new Ω due to stressor intensification and human agency reduction. Watershed SESs are constantly adapting and can enter different AC phases (Fath et al. 2015) when provoked, for example, by large-magnitude shocks that interrupt SES trajectories; this process can lead to a new AC with new conditions from the current phase (Filatova et al. 2016; Gunderson et al. 2017). After the first Ω and entering a new cycle with SES components under new conditions, the CHW entered a long conservation phase related to human agency forces and positive external driver impacts, such as PES programs and NGO programs. The subsequent results can be considered a “coerced regime,” where people artificially hold a system in one state to provide specific benefits through formal and informal institutional management strategies (Winkler et al. 2022).

The loss of positive driver impacts on HES and SES components can lead to collapse and reorganization; these situations are exacerbated by stressors (Filatova et al. 2016) and the loss of external connections (Fath et al. 2015). Human agency, as a positive external driver, can mask negative impacts, but when it decreases, the impact of stressors intensifies (Chaffin et al. 2015). For example, NGO programs, as positive drivers, often have short-term, project-specific funding and do not address the performance tasks needed to maintain adequate conditions. However, weak local ties and a rising technocracy in NGOs can hinder the influence of real change drivers (Banks et al. 2015). Therefore, it is crucial to address these issues to prevent system collapse.

Even if the SES trajectory depends on local conditions, such as urbanization rates (Wang et al. 2021), infrastructure and resource demand (Molle and Wester 2009), or public policies (Räsänen et al. 2019), some general determinants of the change in the SES trajectory can be delineated according to the properties of the drivers that affect them. Moreover, it is interesting to observe the roles of human agency and institutions, as Winkler et al. (2022) noted, and the role of shocks in determining SES trajectories despite local context differences (Rodríguez-Robayo et al. 2020).

### Research contributions and challenges

This research has theoretical and methodological contributions. From a theoretical perspective, it contributes to defining HES as a flow that unites diverse socioecological components of SES, and it describes a system trajectory that contrasts Holling’s patterns. Through the proposed method, which combines qualitative data with the AC framework and the Leopold matrix, it was possible to (1) identify and categorize drivers, as the methodology allows for a comprehensive assessment of various drivers, including natural, social, political, and economic factors, that influence HES and SES components; (2) assess impacts by employing the Leopold matrix, thus enabling a quantitative evaluation of the depth, breadth, and strength of driver impacts on the HES; and (3) track SES trajectories by integrating the AC framework, as doing so enables researchers to analyze the historical trajectory of an SES, identify trajectory phases, and understand the factors that trigger transitions between them. This approach offers a robust framework for understanding the complex interactions between SESs and ecosystem services as a flow; thus, this framework is applicable beyond HESs and coastal watersheds to a wide range of SESs, including urban environments, agricultural landscapes, and forested regions. In addition, understanding the relationships among SES components, drivers, and their impacts on HESs over time is crucial for improving management actions toward sustainability (Locatelli et al. 2017).

However, scholars should consider some adaptations and limitations when applying this methodology. On the conceptual side, assessing trade-offs is especially challenging because of the dynamics of component interdependencies (Galafassi et al. 2017). In terms of ecosystem services, trade-offs are the result of the complex dynamics and nonlinearity of the relationships among both HESs and drivers (Lu et al. 2021). Future research could explore methods to incorporate trade-off analysis into AC frameworks.

The quality and availability of qualitative data can be limitations. Researchers may need to rely on diverse sources, such as interviews, historical records, and local knowledge, to gather sufficient information. A regular limitation is the lack of local knowledge at a larger temporal scale, as noted by Folke et al. (2010). In addition, researchers could add quantitative data, such as weather or hydrological data, which, if they exist, could complement SES trajectory studies (Anderson et al. 2019; Johansson and Abdi 2020). For contextual adaptation, the specific drivers and HESs relevant to a particular SES vary. Therefore, researchers need to tailor the list of drivers and HES indicators to the specific context of their study system.

## Conclusions

In the theoretical and methodological approach proposed in this study, which considers the driver impacts on HESs and the adaptive cycle, the social–ecological watershed historical trajectory was assessed. The results show that the SES does not always comply with a succession process of the AC framework and can change its phases in different ways. For example, over the course of 40 years, the CHW SES has been impacted by natural, political, and social drivers that appear, disappear, or change their intensity in breadth, depth, or strength, influencing the components of the SES and changing its trajectory. Consequently, the SES phases are closely linked to the drivers that characterize them, with shocks, especially external ones, driving either collapse or human agency, each of which can force the SES into a conservation state. The phases were identified as follows: (1) transition from r to Ω, (2) change from Ω to k, and (3) a new Ω. Moreover, while the AC phases were used to characterize the SES and shocks as “tipping points,” further research is needed to focus on when stressors hit thresholds that can trigger system total transformation. By adapting and refining this approach, researchers can gain insights into the resilience, adaptability, and sustainability of SESs, which can help inform management and policy decisions for a more sustainable future.

## Supplementary Information

Below is the link to the electronic supplementary material.

Supplementary file1 (PDF 1319 KB)
