# Supplementary material for: Analysis of the watershed social–ecological system trajectory in Copalita-Huatulco, Mexico: The impact of drivers on hydrological ecosystem services
Source: Ambio. 2024 Sep 3;53(12):1797–812. doi: 10.1007/s13280-024-02064-x (PMC11568105; doi:10.1007/s13280-024-02064-x)
Supplement: Supplementary file 1 — Supplementary file1 (PDF 1319 KB) [file 13280_2024_2064_MOESM1_ESM.pdf]

Ambio

Electronic supplemental material

This supplementary material has not been peer reviewed

Tittle: Analysis of the watershed social–ecological system trajectory in Copalita-Huatulco,

Mexico: The impact of drivers on hydrological ecosystem services

Authors: Angel MERLO-GALEAZZI, V. Sophie AVILA-FOUCAT, Maria PEREVICHTCHIKOVA

APPENDIX 1. SEMI-STRUCTURED INTERVIEW GUIDE

Interviewer presentation

Presentation of objectives: We are investigating the main problems facing the Copalita-Huatulco watershed in Oaxaca, how individuals have faced those problems, and how those problems have changed over time. Among the topics of interest are water services and water governance. Can you answer some questions about these topics? All the information you provide us will remain confidential and will be used only for research purposes. I will show you the information obtained during this interview, and you will be able to comment on it. May I record this conversation?

Section 1. Organizational and personal information

- Can you provide me with your full name?
- Can you tell me about the creation, objectives and actions of your organization?
- In which municipalities/localities of the Copalita-Huatulco watershed do you operate in? How do you approach the towns in which you or operate in prior to starting your work? How long have you been working in those locations?
- What organizations does your institution associate with (or is it affiliated with) to work in Copalita-Huatulco watershed?
- What is your role in the organization? How long have you been a member of that organization?

Section 2. Drivers identification

- What have been the significant events that have impacted the socioecological system in these 40 years, and how have they influenced the dynamics of the community and the natural environment?
- Specifically, in your experience, what are the main problems associated with water?
  - Are these problems recurring?
  - In your opinion, what are the causes of these problems?
    - When did these problems begin?

- ✦ Where did these problems begin or are present?
- ✦ What are the consequences of these problems?
- ✦ How have these problems been resolved?
- ✦ Who has intervened to solve these problems?
- ✦ What are the main institutional challenges in water resource management and problems?

Section 3. Period identification

- Could you identify a specific year in which you perceived a significant change in the dynamics of the social-ecological system being studied? If so, what events or factors do you think contributed to this change and how did they affect the community and environment?
- Is there any milestone or event that you consider to be a turning point in the evolution of the social-ecological system? If so, in what year did it occur and what indicators or signals identified this change in system dynamics?

Section 4. Future

- In your opinion, what is the outlook for water management? What problems will the region face? What kinds of actions would be necessary to prevent or mitigate those problems?
- Whom do you consider to be the most relevant actors in water decision-making in the region?
- Who should make decisions related to water in the region and how?

Conclusion

Our interview has concluded; we thank you very much for the time and information provided. We hope to have the opportunity to collaborate with you on future occasions. I will now stop the recording. Thank you.

APPENDIX 2. CONSULTED LITERATURE FOR DRIVERS DESCRIPTIONS

Driver identified	References
**Alternative tourism**	None
**Aquaculture**	None
**Basin commit**	None
**Bore worm**	None
**Climate change**	Lozano (2013)
**Coffee**	Lozano (2013)
**CONANP programs**	
**Community systems for protected natural areas (CSAP)**	González and Miranda (2004), Jiménez (2005), González et al. (2008)
**Earthquakes**	None
**Fire**	(CONAFOR 2021)
**Gravel extraction**	None
**Huatulco National Park (HNP)**	Cid Rodríguez (2006)
**Huatulco Tourism Complex (HTC)**	Brenner (2005), SECTUR (2019), Talledos Sánchez et al. (2019)
**Hurricane Paulina**	Garcia (2005)
**Hydraulic infrastructure**	INEGI (1990, 2000, 2010, 2020)
**Intensive agriculture**	CONAGUA (2020)
**Livestock**	Lozano (2013)
**Logging**	Lozano (2013)
**Non-governmental Organizations (NGOs)**	Barriga and Nazario (2013)
**Other federal programs**	
**Payment for Ecosystem services (PES)**	CONAFOR (2019)
**Ramsar declaration**	Escalona et al. (2003)
**Roads**	Talledos Sánchez (2012)
**Sewage infrastructure**	INEGI (1990, 2000, 2010, 2020)
**Slash and Burn agriculture**	González-Mora et al. (2006), Lozano (2013)
**Social organization**	Sanchéz (2011)
**Urbanization**	INEGI (1990, 2000, 2010, 2020), CONAGUA (2020)

Cited literature

Barriga, M. L., and A. Nazario. 2013. Evaluación del sistema de pago por servicios ecosistémicos en la cuenca de Copalita-Oaxaca. Xoxocotlán, Oaxaca.: Instituto Tecnólogico del Valle de Oaxaca.

Brenner, L. 2005. State-Planned Tourism Destinations: The Case of Huatulco, Mexico. *Tourism Geographies* 7: 138–164. doi:10.1080/14616680500072349.

Cid Rodríguez, M. C. P. 2006. Interpretación de la realidad socioambiental del Parque Nacional Huatulco para la elaboracion de una propuesta educativa. Maestría, Zapopan, Mexico: Universidad de Guadalajara.

CONAFOR. 2019. *Pago por servicios ambientales*. CONAFOR.

CONAFOR. 2021. Sistema Nacional de Información y Gestion Forestal. *Sistema Nacional de Información y Gestion Forestal*.

CONAGUA. 2020. *Registro Público de Derechos de Agua REPDA*.

Escalona, I., D. Argáez, and M. A. González. 2003. *Ficha informativa de los humedales RAMSAR del sirio Cuencas y corales de la zona costera de Huatulco*. RAMSAR.

Garcia, V. 2005. *La construcción social de riesgos y el huracán Paulina*. Centro de Investigaciones y Estudios Superiores en Antropología Social.

González, M. A., and M. Miranda. 2004. El sistema comunitario para el manejo y protección de la biodiversidad: cuenca Huatulco-Copalita, Oaxaca, México. *LEISA. Revista de agroecología* 19: 7–9.

González, M. A., F. S. Martínez, M. Miranda, I. Martínez, and J. Pérez. 2008. El sistema comunitario para la biodiversidad: Una estrategia para el manejo comunitario del complejo hidrológico Copalita-Zimatán-Huatulco. In *Gestión de cuencas y servicios ambientales. Perspectivas comunitarias y ciudadanas*, ed. R. Paré, D. Robinson, and M. González, 231–258. Mexico, D.F.: SEMARNAT.

González-Mora, I., J. E. Barrios-Ordoñez, and C. G. Leal. 2006. *Problemática ambiental y socioeconómica en las cuencas Copalita, Zimatán y Huatulco, Oaxaca: La perspectiva de los especialistas*. Mexico, D.F.: WWF-México.

INEGI. 1990. *Censo de población y vivienda*. Instituto Nacional de Estadística y Geografía.

INEGI. 2000. *Censo de población y vivienda*. Instituto Nacional de Estadística y Geografía.

INEGI. 2010. *Censo de población y vivienda*. Instituto Nacional de Estadística y Geografía.

INEGI. 2020. *Censo de población y vivienda*. Instituto Nacional de Estadística y Geografía.

Jiménez, J. 2005. *Análisis regional y procesos actuales en las cuencas Copalita, Zimatán y Huatulco.* Programa bosques mexicanos, Sierra Costera de Oaxaca. WWF.

Lozano - 2013 - Plan rector cuenca emblemática Río Copalita.pdf.

Lozano, S. 2013. *Plan rector cuenca emblemática Río Copalita*. SAGARPA.

Sanchéz, L. 2011. El OTC y la construcción de arreglos institucionales a nivel de cuenca: El caso del SICOBI. presented at the Encuentro Nacional de Ordenamiento Territorial Comunutario, November 24, Oaxaca.

SECTUR. 2019. Compendio Estadístico 2019. *Secretaria de Turism*.

Talledos Sánchez, É. 2012. La imposición de un espacio: de La Crucecita a Bahías de Huatulco. *Revista Mexicana de Ciencias Políticas y Sociales* 57. doi:10.22201/fcpys.2448492xe.2012.216.34842.

Talledos Sánchez, É., R. Enríquez, and J. G. Filgueiras. 2019. *Turismo, territorio y política en Bahías de Huatulco, Oaxaca*. México.: Pez en el árbol.

APPENDIX 3. VALUES OF DRIVERS IMPACTS OVER HES COMPONENTS

Driver \ HES Component	Forest Cover	Water Quantity	Hydrology and Flux	Water Quality	Provision Access	Provision Demand	**Total**
Values for drivers impacts on Hydrological Ecosystem Services components for 1985-1996 period							
[illegible]	[illegible]	[illegible]	[illegible]	36	[illegible]	[illegible]	[illegible]
[illegible]	[illegible]	[illegible]	[illegible]	[illegible]	8	[illegible]	[illegible]
[illegible]	18	[illegible]	[illegible]	[illegible]	[illegible]	[illegible]	[illegible]
[illegible]	[illegible]	[illegible]	[illegible]	[illegible]	8	[illegible]	[illegible]
[illegible]	36	[illegible]	[illegible]	[illegible]	[illegible]	[illegible]	[illegible]
[illegible]	18	[illegible]	[illegible]	[illegible]	[illegible]	[illegible]	[illegible]
[illegible]	18	[illegible]	18	[illegible]	[illegible]	[illegible]	[illegible]
Values for drivers impacts on Hydrological Ecosystem Services components for 1997-2000 period							
[illegible]	[illegible]	[illegible]	[illegible]	68.4	[illegible]	[illegible]	[illegible]
[illegible]	[illegible]	[illegible]	[illegible]	[illegible]	9.6	[illegible]	[illegible]
[illegible]	18	[illegible]	[illegible]	9	[illegible]	[illegible]	[illegible]

HTC	-19.8	-19.8	-19.8	-9.9	8.8	-2.2	**-62.7**
Coffee	28.8			-14.4			**14.4**
Alternative Tourism	21.6					-2.4	**19.2**
NGO	21.6		21.6				**43.2**
Intensive Agr.	-21.6	-21.6		-10.8			**-54**
CSAP	18		18				**36**
Earhquakes	-180	-180			-40		**-400**
Hurricane Paulina	-180	-180	-180	-90	-80		**-710**
HNP	18		18		-8		**28**
Fire	-43.2						**-43.2**
Total	-416.7	-423	-209.7	-172.8	-109.6	-7.7	**-1339.5**

Values for drivers impacts on Hydrological Ecosystem Services components for 2001-2014 period

				86.4			
					8		
	18			9			
					15.2		
	21.6						
	25.2						
	54			27	12		
	43.2		21.6				
	18	18	18	18			
			9				

CONANP Programs	36				8		**44**
Others federal programs	36	18		9	4		**67**
Basin comIt	36			36			**72**
PES	36						**36**
Climate Change		-36	-36		-4		**-76**
**Total**	**43.36**	**-77.4**	**-106.2**	**-54.9**	**35.2**	**-11.2**	**-171.14**

Values for drivers impacts on Hydrological Ecosystem Services components for 2015-2020 period

				93.6			
					20.8		
	28.8			7.2			
	21.6	21.6		10.8			
	50.4						
	57.6	14.4		14.4			
	43.2		43.2				
			9				
	18	18	18	18			
	1.8						
